# EGFR-D770>GY and Other Rare EGFR Exon 20 Insertion Mutations with a G770 Equivalence Are Sensitive to Dacomitinib or Afatinib and Responsive to EGFR Exon 20 Insertion Mutant-Active Inhibitors in Preclinical Models and Clinical Scenarios

**DOI:** 10.3390/cells10123561

**Published:** 2021-12-17

**Authors:** Ikei S. Kobayashi, Hollis Viray, Deepa Rangachari, Susumu S. Kobayashi, Daniel B. Costa

**Affiliations:** 1 Department of Medicine, Division of Medical Oncology, Harvard Medical School, Boston, MA 02215, USA; ikobayas@bidmc.harvard.edu (I.S.K.); hviray@bidmc.harvard.edu (H.V.); drangach@bidmc.harvard.edu (D.R.); skobayas@bidmc.harvard.edu (S.S.K.); 2 Exploratory Oncology Research and Clinical Trial Center, National Cancer Center, Division of Translational Genomics, Kashiwa 277-8577, Japan

**Keywords:** lung cancer, EGFR exon 20 insertion, D770>GY, afatinib, dacomitinib, poziotinib, mobocertinib

## Abstract

*Epidermal growth factor receptor* (*EGFR*) exon 20 insertion mutations account for a tenth of all *EGFR* mutations in lung cancers. An important unmet clinical need is the identification of EGFR exon 20 insertion mutants that can respond to multiple classes of approved EGFR-TKIs. We sought to characterize variants involving *EGFR*-D770 to *EGFR*-G770 position equivalence changes that structurally allow for response to irreversible 2nd generation EGFR-TKIs. Our group used preclinical models of EGFR exon 20 insertion mutations to probe representative 1st (erlotinib), 2nd (afatinib, dacomitinib), 3rd generation (osimertinib) and EGFR exon 20 insertion mutant-active (poziotinib, mobocertinib) TKIs; we also queried the available clinical literature plus our institutional database to enumerate clinical outcomes. *EGFR*-D770>GY and other *EGFR* insertions with a G770 equivalence were identified at a frequency of 3.96% in separate cohorts of *EGFR* exon 20 insertion mutated lung cancer (*n* = 429). Cells driven by EGFR-D770>GY were insensitive to erlotinib and osimertinib, displayed sensitivity to poziotinib and mobocertinib and were uniquely sensitive to afatinib and dacomitinib in comparison with other more typical EGFR exon 20 insertion mutations using proliferation and biochemical assays. Clinical cases with *EGFR*-G770 equivalence from the literature and our center mirrored the preclinical data, with radiographic responses and clinical benefits restricted to afatinib, dacomitinib, poziotinib and mobocertinib, but not to erlotinib or osimertinib. Although they are rare, at <4% of all exon 20 insertion mutations, EGFR-G770 equivalence exon 20 insertion mutations are sensitive to approved 2nd generation EGFR TKIs and EGFR exon 20 insertion mutant-active TKIs (mobocertinib and poziotinib). EGFR-D770>GY and other insertions with a G770 equivalence join EGFR-A763_Y764insFQEA as exon 20 insertion mutationsresponsive to approved EGFR TKIs beyond mobocertinib; this data should be considered for clinical care, genomic profiling reports and clinical trial elaboration.

## 1. Introduction

The most heterogeneous group of kinase domain *epidermal growth factor receptor* (*EGFR*) mutations comprise ten percent of cases with in-frame insertions within exon 20 [1,2]. The majority of these mutants cluster within a structural position following the regulatory C-helix of EGFR, activate auto-phosphorylation without significant changes within the ATP binding kinase domain and have been shown not to generate a therapeutic window to the initial wave of approved EGFR tyrosine kinase inhibitors—including gefitinib, erlotinib, afatinib, dacomitinib and osimertinib [2,3,4,5,6,7]. Over the last half-decade, the structure-based drug development of novel EGFR TKIs with a therapeutic window against these more common EGFR exon 20 insertion mutants has led to the clinical development and regulatory approval of mobocertinib [7,8]. Outside of EGFR TKIs, the bi-specific EGFR antibody amivantamab-vmjw has shown some preclinical activity against multiple EGFR mutated lung cancer models and was also approved for use in advanced *EGFR* exon 20 insertion mutated lung cancer [9]. The clinical activity of both mobocertinib and amivantamab is relatively modest in view of dose-limiting adverse events and inadequate pharmacokinetic-pharmacodynamic parameters [8,9,10].

However, not all EGFR exon 20 insertion mutants are insensitive to clinically available EGFR TKIs outside of mobocertinib. The unique EGFR-A763_Y764insFQEA mutant is pan-sensitive to all classes of EGFR TKIs in preclinical models, and patients with tumors harboring this genomic change have responded to gefitinib, erlotinib, afatinib, osimertinib, poziotinib, CLN-081 and mobocertinib [2,5,6,7].

**Figure 1:**
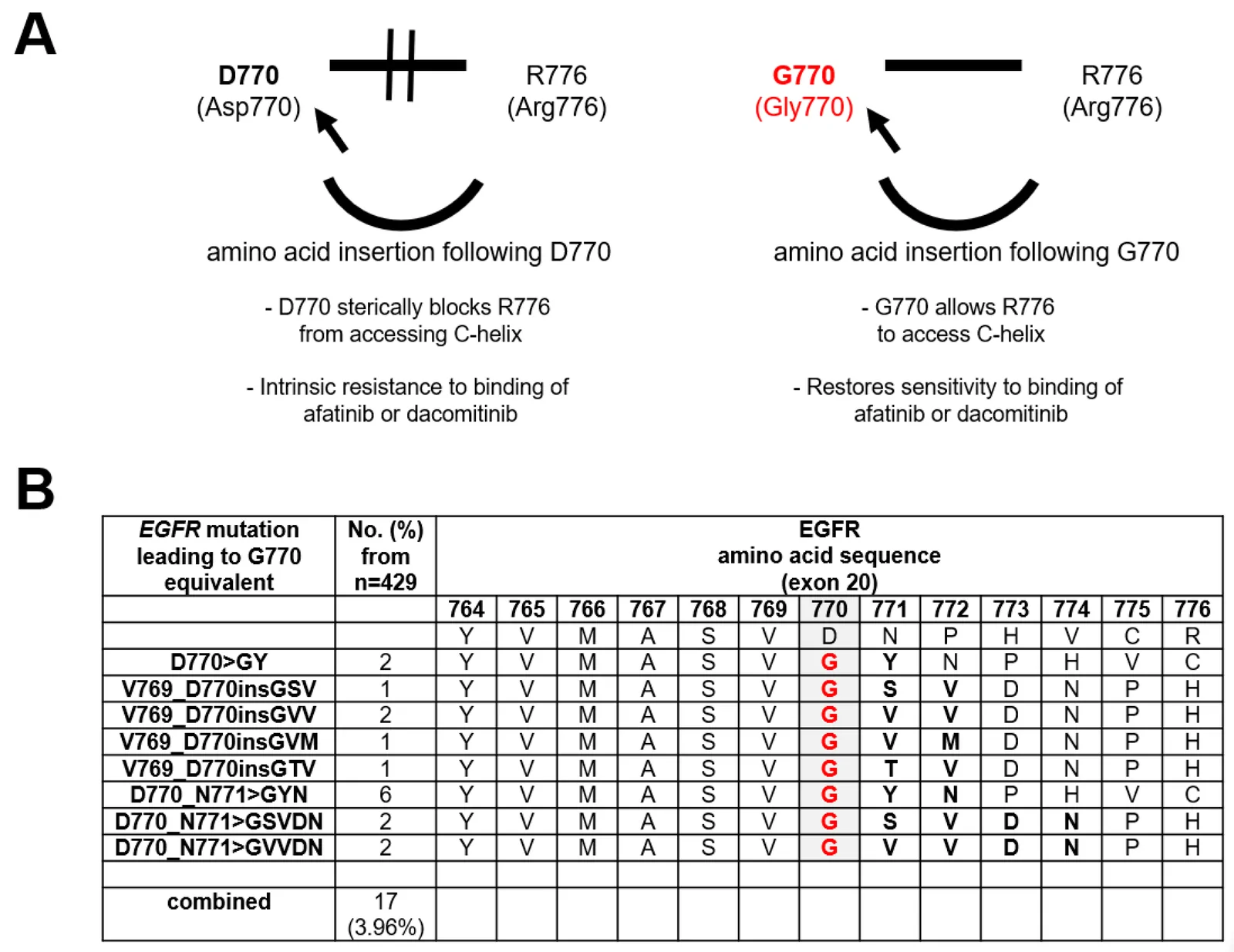
EGFR mutations with a D770 to G770 equivalent change. (**A**) Structural-model basis of the sensitivity of EGFR-D770>GY and other exon 20 insertion mutations with a G770 equivalence. The model given in [11] details the interactions that may allow for sensitivity to reversible 2nd generation EGFR TKIs and, purportedly, to other inhibitors. (**B**) Frequency of *EGFR* exon 20 insertion mutations in three separate cohorts of *EGFR* mutated non-small-cell lung cancer, obtained from [1,11,12], with a total of 429 *EGFR* exon 20 insertion mutated lung cancer cases identified.

Few other variants with sensitivity to 1st, 2nd or 3rd generation EGFR TKIs have been reported. We sought to characterize a less frequent group of EGFR exon 20 insertions that are associated with structural kinase changes occurring when the aspartate amino acid at position 770 (D770) is replaced by a glycine (G770) or an equivalent structural change, leading to conformational shifts that, in the context of insertions, allow for sensitivity to irreversible 2nd generation EGFR inhibitors [11] such as afatinib and dacomitinib (Figure 1A). 

We generated the most comprehensive set of preclinical studies of EGFR TKIs in this type of mutant and compiled the largest cohort of clinical cases with genotype-EGFR TKI response for tumors harboring EGFR-D770>GL or other mutations leading to G770 equivalence.

## 2. Materials and Methods

### 2.1. Drugs

Erlotinib, afatinib, osimertinib (LC Laboratories Woburn, MA, USA), dacomitinib (Selleckchem [Houston, TX, USA]), poziotinib (AdooQ BioScience Irvine, CA, USA) and mobocertinib (MedChemExpress, Monmouth Junction, NJ, USA) were dissolved in dimethyl sulfoxide and stored at −80 °C. 

### 2.2. Preclinical Models/Cell Lines

Ba/F3 murine cell lines were maintained in RPMI 1640 medium (Corning) supplemented with 10% fetal bovine serum. In the case of EGFR-WT driven Ba/F3 cells, 10 ng/mL of EGF was added. All cells were grown at 37 °C in a humidified atmosphere with 5% CO_2_ and tested for absence of mycoplasma contamination (MycoAlert Mycoplasma Detection Kit, Lonza Basel, Switzerland) prior to experiments (initiated within the initial 1 to 4 passages). 

### 2.3. Generation of EGFR-D770>GY Mutation

The *EGFR*-D770>GL (delD770insGY) mutation was introduced into the *EGFR*-WT sequence construct in the context of the MigR1 retrovirus vector (Addgene [Watertown, MA, USA]) using the Q5^®^ Site-Directed Mutagenesis Kit (New England BioLabs [Ipswich, MA, USA]) as published for other Ba/F3 mutations [2,6,7]. Details of the expression of EGFR mutants in Ba/F3 preclinical models and of the generation of these systems for TKI dose-response experiments can be found in our prior publications [2,6,7].

### 2.4. Proliferation Assays

Cell viability was determined using the CellTiter 96 aqueous one solution proliferation kit (Promega, Madison, WI, USA) for Ba/F3 and other cells. Cells were plated in 96-well plates and then treated in the appropriate medium, with or without EGFR TKIs, for 3 days. Inhibitory proliferation curves and the 50% inhibitory concentration (IC_50_) were generated using GraphPad Prism (version 8, GraphPad Software, San Diego, CA, USA). 

### 2.5. Protein-Level Analysis

Western blot lysates and preparation were performed as previously described [2,10]. Total EGFR, β-actin antibodies (Santa Cruz Biotechnology, Dallas, TX, USA) and phospho-EGFR (pY1068) antibody (ThermoFisher, Waltham, MA, USA) were at 1:1000 dilution, while secondary antibodies were at 1:5000 dilution.

### 2.6. Patient-Level Data Collection

The frequency of *EGFR* exon 20 insertion mutations was calculated using three separate cohorts of cases [1,11,12]. One novel clinical, radiographic and survival outcome used for this study was obtained from an ongoing Institutional Review Board approved protocol at our institution. Additional genotype-inhibitor data were obtained through a literature review of studies published in PubMed and other databases, as well as oncology meeting abstracts, using the search terms “EGFR insertion exon 20” + “D770”. A total of 11 articles/abstracts had data on *EGFR*-D770>GY and other rare *EGFR* exon 20 insertions with a G770 equivalence and EGFR TKI use [2,11,12,13,14,15,16,17]. Response evaluation criteria in solid tumors (RECIST) was used, when provided. Progression-free survival (PFS) and overall survival (OS) were calculated in months, from time of initiation of an EGFR TKI, when provided.

## 3. Results 

### 3.1. Frequency of EGFR Exon 20 Insertions with a G770 Equivalence

We queried three separate cohorts of EGFR exon 20 insertion mutations [1,11,12]. Out of the 429 cases reported, 17 (3.96%) had the *EGFR* mutation leading to G770 equivalent change in the context of an insertion (Figure 1B). The types of alterations varied from indels—such as D770>GY—to complex insertions of three to five amino acids (Figure 1B). 

### 3.2. Preclinical Characterization of an EGFR Exon 20 Insertion Mutant with a G770 Equivalence

**Figure 2:**
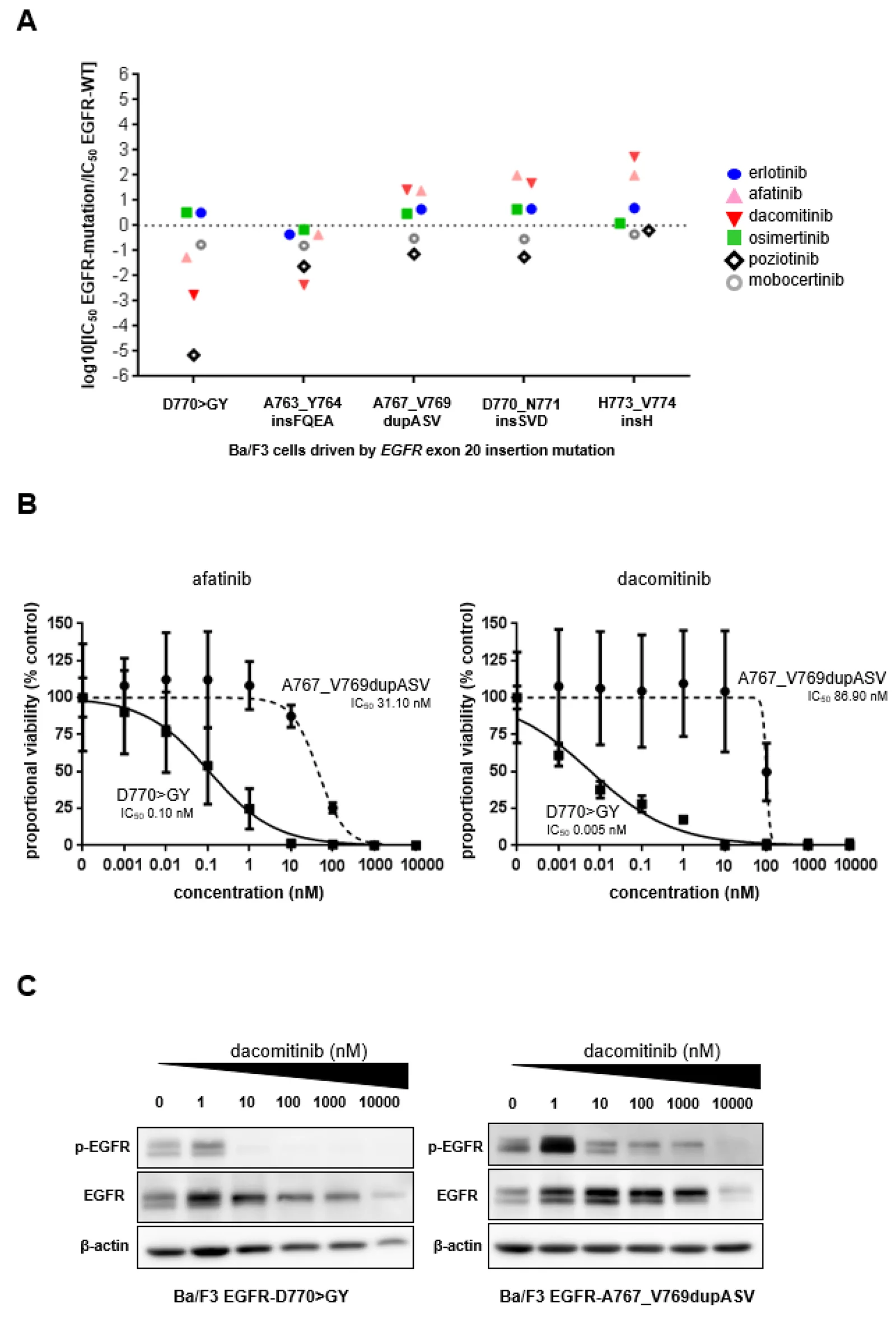
Ba/F3 system isogenic preclinical models of EGFR exon 20 insertions mutations to probe EGFR-TKIs. (**A**) Therapeutic window of different EGFR-TKIs to a set of EGFR exon 20 mutants. Cells were plated at a density of 10,000 cells per well (96-well plates) and grown over 3 days after treatment. Logarithm of the 50% inhibitory concentration (IC_50_) of EGFR exon 20 mutants compared to EGFR-WT is plotted with 3 separate experiments used to generate IC_50_. Values below zero (0) indicate sensitivity, while values above 0 indicate resistance to EGFR-TKIs. The therapeutic window of Ba/F3 cells with *EGFR*-D770>GY are contrasted with other exon 20 insertion mutations. (**B**) Dose-response proliferation assays (the proportional percent viability) of dacomitinib and afatinib for cells with *EGFR*-D770>GY compared with those with *EGFR*-A767_V769dupASV. Three separate experiments were used to generate IC_50_, and standard deviations are depicted in vertical bars. For afatinib, the 95% confidence intervals (95%CIs) did not overlap for both mutants with values of IC_50_ 31.10 nM (95%CI 20.37–47.47) and 0.10 nM (95%CI 0.07–0.15). For dacomitinib, the 95% CIs did not overlap for both mutants with values of IC_50_ 86.90 nM (95%CI 65.43–115.40) and 0.005 nM (95%CI 0.002–0.016). (**C**) Western blotting of Ba/F3 cells driven by EGFR-D770>GY and EGFR-A767_V769dupASV mutants. Cells were treated with the EGFR-TKI dacomitinib for 8 h at the indicated ascending concentrations. pEGFR, phosphorylated EGFR at position 1068, total EGFR and β-actin as a loading control are displayed in the graphical compilation.

Our group generated a Ba/F3 cell line driven by the EGFR-D770>GY mutant in order to compare its properties with our previously described isogenic Ba/F3 preclinical models of exon 20 insertion mutants (Figure 2). To evaluate the preclinical therapeutic window of these cells to various EGFR TKIs, we used proliferation assays to contrast the inhibitory concentrations of these drugs towards each mutant against the EGFR-WT. Ba/F3 cells with *EGFR*-D770>GY were uniquely sensitive to 2nd generation irreversible TKIs, afatinib and dacomitinib (Figure 2A). These cells also had a favorable therapeutic window to mobocertinib and poziotinib (Figure 2A). The EGFR-D770>GY mutant-driven cells were insensitive to doses of erlotinib (1st generation reversible) and osimertinib (3rd generation covalent EGFR TKI) that spared the EGFR-WT (Figure 2A). As detailed in our prior work [2,6,7], cells with the *EGFR*-A763_Y764insFQEA mutation were pan-sensitive to all EGFR TKIs tested; cells with more common EGFR exon 20 insertion mutations (*EGFR*-A767_V769dupASV, D770_N771insSVD, N773_V774insH) were insensitive to erlotinib, afatinib, dacomitinib and osimertinib, but sensitive to mobocertinib and poziotinib (Figure 2A). 

To highlight the differences in proliferation assays between Ba/F3 cells driven by the EGFR-D770>GY mutant and the more typical EGFR-A767_V769dupASV mutant, we show the dose-response curve for increasing concentrations of afatinib or dacomitinib (Figure 2B). While cells with the A767_V769dupASV mutant had an IC_50_ that exceeded 30 nM for these 2nd generation EGFR TKIs, the cells with D770>GY had an IC_50_ below 0.1 nM (Figure 2B). 

The exquisite sensitivity to 2nd generation EGFR TKIs was confirmed at the biochemical level. In Western blot experiments, the phosphorylated form of EGFR was readily inhibited by 10 nM and higher doses of dacomitinib in the EGFR-D770>GY mutant (Figure 2C). The same level of inhibition of phosphorylated EGFR was only achieved by 1000 nM of dacomitinib in the EGFR-A767_V769dupASV mutant (Figure 2C). Similar data were obtained with afatinib (data not shown). 

Our aforementioned preclinical results confirmed the structural modeling of EGFR-D770>GY (Figure 1A) and led us to speculate that patients with advanced lung cancers harboring *EGFR* exon 20 insertion mutations with a G770 equivalence could respond to afatinib, dacomitinib, poziotinib and mobocertinib, but would not derive clinical benefit from gefitinib, erlotinib or osimertinib. 

### 3.3. Clinical Outcomes of Reported Patients with Advanced Lung Cancers Harboring EGFR Exon 20 Insertion Mutations Encompassing G770 Equivalence

We identified seven reports from the literature and added one case from our institutional cohort that detailed partial clinical-radiographic parameters in patients with metastatic lung cancers harboring *EGFR* exon 20 insertion mutations encompassing G770 equivalence, in which an EGFR TKI was provided (Table 1). Out of eleven separate cases, three received a 1st generation EGFR TKI (erlotinib), two received a 2nd generation EGFR TKI (dacomitinib, afatinib), one received a 3rd generation EGFR TKI (osimertinib) and five received an exon 20 active EGFR TKI (mobocertinib, poziotinib). It is interesting, and congruent with our preclinical studies (Figure 2A), that patients with these tumors did not respond to or derive clinical benefit from erlotinib or osimertinib (Table 1). 

Radiographic responses and prolonged periods of clinical benefit (exceeding 10 months) were seen in the two cases where dacomitinib or afatinib were used (Table 1). 

The majority—but not all—of cases that received poziotinib or mobocertinib in this compiled cohort of advanced lung cancers harboring *EGFR* exon 20 insertion mutations with G770 equivalence had radiographic responses (Table 1). In the case of mobocertinib, the duration of response exceeded one year (Table 1). 

Although these cases are limited in number and by reporting biases, they provide supporting evidence that EGFR-D770>GY and other exon 20 insertion mutations with G770 equivalence are sensitive to the clinically available EGFR TKIs dacomitinib, afatinib and mobocertinib. 

## 4. Discussion

Our combined preclinical and clinical results provide robust characterizations of EGFR-D770>GY and other EGFR exon 20 insertion mutants with a resulting G770 equivalent change, which comprise approximately 4% of all EGFR exon 20 mutations in lung cancer. The initial structure-based characterization of the G770 equivalence change in the context of exon 20 insertion mutations occurred in 2017 [11]. These amino acid insertions within exon 20 activate EGFR in a fashion similar to other more common EGFR exon 20 insertion mutations [2]. However, the equivalent G770 change allows for an interaction with amino acid R776, which allows access to the C-helix and restores sensitivity to irreversible EGFR TKIs [11] such as afatinib or dacomitinib (Figure 1A). These initial structural-biochemical results did not evaluate the diversity of EGFR TKIs available or in development in 2021. The current report provides a preclinical drug-response categorization of all clinically available classes of EGFR TKIs—1st, 2nd, 3rd and exon 20 insertion active kinase inhibitors against EGFR-D770>GY (Figure 2) and the most comprehensive description of clinical outcomes of patients with tumors harboring *EGFR* exon 20 insertion mutations with G770 equivalence treated with EGFR TKIs (Table 1). 

EGFR-D770>GY has a therapeutic window to the clinically approved EGFR exon 20 insertion mutation active EGFR TKI mobocertinib and the in-development TKI poziotinib, mirroring a pattern similar to that of more typical EGFR exon 20 insertion mutations, which retain D770 at the structural level, such as A767_V769dupASV, D770_N771insSVD and H773_V774insH (Figure 2A). The clinical trials that support the development of mobocertinib and poziotinib enrolled cases with tumors containing EGFR exon 20 insertion mutations with G770 equivalence [8,15,17]. We have been able to highlight radiographic responses and prolonged periods of clinical benefit in most but not all treated cases (Table 1). Mobocertinib (160 mg daily) and poziotinib (16 mg daily) are EGFR TKIs with a narrow therapeutic window in the clinic with the majority of patients experiencing moderate to severe gastrointestinal and cutaneous adverse events that can limit their clinical impact [8,15]. The reported radiographic response rates in patients with non-small-cell lung cancer with *EGFR* exon 20 insertion mutations range from less than 15% for poziotinib to close to 30% for mobocertinib but the duration of response can exceed 15 months [8,10,15,17]. 

EGFR-D770>GY is different than other typical EGFR exon 20 insertion mutations that retain D770 at the structural level since it is exquisitely sensitive to afatinib and dacomitinib. The therapeutic window to these irreversible 2nd generation EGFR TKIs numerically exceeded the window seen with the EGFR TKI pan-sensitive EGFR exon 20 insertion mutation A763_Y764insFQEA (Figure 2A). We were also able to identify two clinical cases with clinical-radiographic benefit from the use of dacomitinib or afatinib (Table 1). Afatinib is currently only approved for use in tumors with *EGFR*-exon 19 deletions and L858R, L861Q, G719X and S768I mutations, while dacomitinib’s approval is limited to tumors with *EGFR*-exon 19 deletions and L858R mutation [18,19,20]. Both of these EGFR TKIs are potent EGFR-WT inhibitors and are fraught with cutaneous, gastrointestinal and mucosal adverse events during clinical use that often require dose reductions [18,20]. Other groups have consistently shown in preclinical models that EGFR-D770>GY is inhibited by clinically achievable concentrations of 2nd generation EGFR TKIs [21,22]. The aforementioned data can be used to support the off-label use of dacomitinib or afatinib in selected EGFR TKI-naïve cases of advanced lung cancers harboring *EGFR* exon 20 insertion mutations with a G770 equivalent change. It has become evident in 2021 that a structure-based approach for defining functional groups of EGFR mutations is a more robust method to define EGFR TKI response patterns than traditional exon based classifications [22]. Efforts to provide preclinical structure to inhibitor profiles that can be validated in clinical cohorts will undoubtedly improve the selection of EGFR TKIs in the clinic, especially for less common mutations [22].

The limitations of our study include the lack of in vivo preclinical models with which to test EGFR antibodies or antibody-drug conjugates under development for EGFR exon 20 insertion mutations [9,10]; the limited number of clinical cases identified by our literature search; the incomplete data on outcomes with novel EGFR TKIs; and the lack of characterizations of putative on-target or off-target mechanisms of resistance to EGFR TKIs. These experiments and more robust clinical cohorts will need to be explored in future work. In addition, the close structural similarity of exon 20 of EGFR and receptor tyrosine-protein kinase erbB-2 (ERBB2) indicates that our findings could be applicable to *ERBB2* mutated lung cancer [10]. Rare ERBB2 exon 20 insertion mutants with a similar shared glycine amino acid [11], such as ERBB2-M774>WLV, ERBB2-G778_S779insCPG or ERBB2-P780_Y781insGSP, may be equally susceptible to afatinib, dacomitinib, mobocertinib and poziotinib. 

In summary, we provide the most detailed available portrayal of a rare group of *EGFR* exon 20 insertion mutations with a G770 equivalent change. These mutants are sensitive to the recently approved EGFR TKI mobocertinib and the in-development EGFR TKI poziotinib, and they are exquisitely sensitive to the reversible EGFR TKIs dacomitinib or afatinib.

## Figures and Tables

**Table 1:** Outcomes of reported patients with advanced/metastatic lung cancers harboring *EGFR* exon 20 insertion mutations encompassing G770 equivalence.

*EGFR* Mutation	EGFR TKI and Dose	Response by RECIST	RECIST % Change	PFS/TTD	OS	Reference
D770>GY	erlotinib 150 mg/day	PD	+38.1	1 month	12 months	[1]
D770>GY	erlotinib 150 mg/day	PD	+6%	1 month	1.5 months	[1]
D770_N771>GSVDN	erlotinib NR dose	PD	NR	1.5 months	NR	[12]
D770>GY	dacomitinib 45 mg/day	PR	NR	12.4 months	NR	[14]
D770>GY	afatinib NR dose *	PR	NR	11 months	NR	[13]
D770>GY	osimertinib 160 mg/day	PD	+39%	2 months	3 months	current report
D770>GY	poziotinib 16 mg/day	PR	−30%	NR	NR	[15]
V769_D770insGSV	poziotinib 16 mg/day	SD	−5%	NR	NR	[15]
D770>GY	mobocertinib 160 mg/day	PD	+25%	2 months	NR	[8]
D770>GY	mobocertinib 160 mg/day	PR	NR	12+ months	12+ months	[17]
V769_D770insGG	mobocertinib 160 mg/day	PR	NR	18+ months	18+ months	[17]

* afatinib was provided in conjunction with cetuximab. EGFR, epidermal growth factor receptor; TKI, tyrosine kinase inhibitor; RECIST, response evaluation criteria in solid tumors; PFS, progression-free survival; TTD, time to treatment discontinuation; OS, overall survival; PD, progressive disease; PR, partial response; SD, stable disease; NR, not reported; +, ongoing response/time frame.

## Data Availability

The original contributions presented in this study are included in the article. Further inquiries can be directed to the corresponding author.
